# Short-Term Fish Oil Treatment Changes the Composition of Phospholipids While Not Affecting the Expression of Mfsd2a Omega-3 Transporter in the Brain and Liver of the 5xFAD Mouse Model of Alzheimer’s Disease

**DOI:** 10.3390/nu10091250

**Published:** 2018-09-06

**Authors:** Desanka Milanovic, Snjezana Petrovic, Marjana Brkic, Vladimir Avramovic, Milka Perovic, Sanja Ivkovic, Marija Glibetic, Selma Kanazir

**Affiliations:** 1 Department of Neurobiology, Institute for Biological Research, University of Belgrade, Bulevar despota Stefana 142, 11060 Belgrade, Serbia; marjana.brkic@ibiss.bg.ac.rs (M.B.); vladimir.avramovic@ibiss.bg.ac.rs (V.A.); milkap@ibiss.bg.ac.rs (M.P.); sanja.ivkovic@ibiss.bg.ac.rs (S.I.); 2 Center of Excellence in Nutrition and Metabolism Research, CENM, Tadeusa Koscuska 1, 11000 Belgrade, Serbia; snjezana570.imr12@gmail.com (S.P.); mglibetic@gmail.com (M.G.)

**Keywords:** Mfsd2a, docosahexaenoic acid, Alzheimer’s disease, *n*-3 polyunsaturated fatty acids, amyloid-β, *n*-6 fatty acids

## Abstract

Long-term fish oil (FO) supplementation is able to improve Alzheimer’s disease (AD) pathology. We aimed to determine the impact of short-term fish oil (FO) intake on phospholipids composition and plaque pathology in 5xFAD mice, a widely used animal model of AD. A 3-week-long FO supplementation administered at 3 months of age decreased the number of dense core plaques in the 5xFAD cortex and changed phospholipids in the livers and brains of wild-type (Wt) and 5xFAD mice. Livers of both genotypes responded by increase of *n*-3 and reciprocal decrease of *n*-6 fatty acids. In Wt brains, FO supplementation induced elevation of *n*-3 fatty acids and subsequent enhancement of *n*-6/*n*-3 ratio. However, in 5xFAD brains the improved *n*-6/*n*-3 ratio was mainly due to FO-induced decrease in arachidonic and adrenic *n*-6 fatty acids. Also, brain and liver abundance of *n*-3 fatty acids were strongly correlated in Wts, oppositely to 5xFADs where significant brain-liver correlation exists only for *n*-6 fatty acids. Expression of omega-3 transporter Mfs2a remained unchanged after FO supplementation. We have demonstrated that even a short-term FO intake improves the phospholipid composition and has a significant effect on plaque burden in 5xFAD brains when applied in early stages of AD pathology.

## 1. Introduction

The polyunsaturated fatty acids (PUFA) of omega-3 (*n*-3) and omega-6 (*n*-6) series are essential for prenatal brain development and the normal maintenance of brain functions and vision in adults. Since de novo synthesis of PUFA is very low within the brain, the blood must supply PUFA to the brain either from exogenous PUFA obtained through diet or from endogenous liver synthesis from dietary precursors [1]. In the context of the modern human lifestyle and diet, an absolute change of omega-6/omega-3 in the food supply of Western societies has occurred over the last 150 years, raising a ratio of omega-6/omega-3 to 20:1 instead of 1:1 as was in our ancestral diets [2]. An unbalanced omega-6/omega-3 ratio plays an important role in the development of many chronic and inflammatory conditions like coronary disease, diabetes, arthritis, cancer and neurological and psychiatric diseases. As omega-3 PUFA supplementation was shown to play a significant role in the prevention and management of some of these conditions, in recent years omega-3 has been considered of a particular interest for its potential to ameliorate or delay the onset of Alzheimer’s disease (AD). 

Numerous studies have demonstrated that omega-3 supplementation, mainly performed through dietary or oral administration of docosahexaenoic acid (DHA) is able to improve AD-associated brain pathology. Thus in different transgenic mouse models of AD, omega-3 supplementation was shown to reduce amyloid plaque burden [3,4,5,6,7,8,9,10], reduce Thau pathology [6,11], improve learning and memory [4,5,7,9,12] decrease inflammation [4,13,14], reduce neuronal loss and neurodegeneration [7,12,15], and improve neurogenesis and synaptic plasticity [16]. Similar effects of omega-3 supplementation were confirmed in a rat model of AD caused by amyloid-β injections [15] and also in a 3xTG-AD/Fat-1 mouse that can convert endogenous omega-6 to omega-3 [17]. Synthetic hydroxylated derivatives of DHA was also shown to have beneficial effects on AD hallmarks [18,19,20], as well as fish oil, a natural source of omega-3 fatty acids [21,22]. These findings in animal models are supported by epidemiological studies on humans, revealing the improvement of mild cognitive impairment by omega-3 treatment and an inverse relation between omega-3 intake and AD incidence, although some discordant findings still exist [1,23]. 

Despite the fact that PUFAs, especially DHA, are very abundant in the phospholipids of the neural membrane, the mechanisms of their uptake into the brain are not well understood. Extensive presence of fatty acids (FA) transporters and binding proteins indicate that they can be critically involved in the intake and enrichment of PUFAs, although it is still unclear to what extent simple diffusion contributes to the total rate of PUFAs intake [24]. Thus, of great importance was the recent finding of Nguyen et al. [25] who identified Mfsd2a Mfsd2a, previously an orphan transporter, as the major transporter for DHA uptake in the brain. Mfsd2a plays an essential role in maintaining DHA levels during embryogenesis and in establishing the integrity of the blood–brain barrier (BBB). Genetic ablation of Mfsd2a results in neurovascular dysfunction due to BBB leakage, as seen in neurodegenerative disorders and AD [25,26]. Mfsd2a can be induced in mouse liver by fasting or a high-fat diet, indicating a potential physiological role in lipid metabolism and energy expenditure [27,28], but its expression in the presence of excessive DHA has not been evaluated. 

Previous examination of post-mortem autopsy brain samples found significant DHA reduction in AD patients that may be related to cognitive decline [1,23,29] and some clinical trials revealed a certain recovery of cognitive capacity in patients with mild to moderate AD who were administered omega-3 from fish oil [23]. Studies on animals demonstrated the ability of supplementation with omega-3, DHA, DHA and EPA, or DHA derivate to change the brain fatty acid composition and to significantly increase levels of DHA and total omega-3 PUFAs accompanied by a reciprocal decrease of individual and total omega-6 fatty acids [3,5,7,12,21,30,31,32]. These changes can modify cellular membrane structures and reduce oxidative modifications [33,34] since phospholipids with a higher percentage of DHA have greater flexibility and less ordered packing of hydrocarbon chains that can alter lateral movement of proteins, ion channels, and detergent-insoluble lipid rafts. Amyloidogenic APP (amyloid precursor protein) processing, aside from intracellular compartments, is believed to occur in lipid rafts of the synaptic membrane, the sites where APP protein and β- and γ-secretases are located. Changes in brain DHA concentration could affect lateral mobility collision rates between APP and secretases thus down-regulating APP processing and deposition of amyloid beta (Aβ) cleavage product into plaques, hallmarks of AD disease [8,31,35]. Also, accumulating evidence indicates that Aβ plays an important role in regulating lipid homeostasis, either by direct effect or influencing gene expression/stability of enzymes involved in lipid synthesis, suggesting bidirectional link between APP and lipids [36]. 

Although 5xFAD is a widely used mouse model of AD, to the best of our knowledge, there are only few studies that have investigated the effects of omega-3 supplementation in this mouse model. They used DHA [4] or DHA synthetic derivate HDHA [18,20,31] and showed elevated very long-chain fatty acids (>22 carbons) and reduced Aβ production [18,20,31]. A comprehensive examination of the long-chain (≥16 carbons) fatty acids in the brain and liver of 5xFAD mice following short-term fish oil (FO) treatment implemented during the early phase of AD pathology, prior to the extensive plaque depositions, has not been undertaken previously. Therefore, in this study we aimed (1) to characterise the lipid profile of 5xFAD mice in comparison to wild-type littermates, (2) to examine whether very short FO supplementation affects the fatty acid composition of brain phospholipids, (3) to examine if FO supplementation affects Mfsd2a expression, and (4) to evaluate the effects of FO on the amyloid plaque burden in the cortex of transgenic animals. As previous evidence suggested the importance of peripheral omega-3 fatty acids, we also performed a lipid analysis of the liver, due to its indispensable contribution in supplying DHA and other essential fatty acids to the brain. 

## 2. Materials and Methods

### 2.1. Animals

In this study we used the 5xFAD mouse model, bearing five familial AD human mutations (3 in APP gene–*Swedish*, *Florida*, and *London*, and two in PS1 gene-*M146L* and *L286V*) under transcriptional control of the neuron-specific murine Thy-1 promoter, as described by Oakley et al. [37]. 5XFAD transgenic male mice were crossed with C57BL/6xSJL female mice (both obtained from Jackson Laboratory, Bar Harbor, ME, USA). The resulting offspring (heterozygous 5xFAD transgenic and non-transgenic, wild-type littermates as their controls) were used in experiments. Genotyping was performed by polymerase chain reaction (PCR) of tail DNA according to the supplier’s protocol. The animals (3–4 mice per cage) were housed under standard conditions (23 ± 2 °C, 60–70% relative humidity, 12 h light/dark cycles, free access to food and water). All animal procedures were in compliance with the Directive (2010/63/EU) on the protection of animals used for experimental and other scientific purposes and was approved by the Ethical Committee for the Use of Laboratory Animals (resolution No. 01-06/13) of the Institute for Biological Research, University of Belgrade. Minimal numbers of animals were used and all efforts were made to minimize animal suffering.

### 2.2. Treatments

All animals were raised on commercial rodent chow (diet composition and body weights are given in the Table 1 and Table 2, respectively). At 3 months of age, animals were divided into 4 groups: control, wild-type and transgenic groups (Wt, *n* = 10, and 5xFAD, *n* = 12, respectively) and treated, FO-supplemented Wt and transgenic groups (Wt FO, *n* = 10, and 5xFAD FO, *n* = 12, respectively). The 100 µL of the fish oil (fatty acid composition in Table 3) was administered to the treated groups for 21 days via oral gavage. The control groups received the same volume of the water as vehicle, Although other oils can be used as a control for the fish oil treatment, as dietary FO contains other fatty acids (apart from omega-3 and omega-6 fatty acids), iodine, furan fatty acids, as well as anti-oxidant vitamin E that can have a certain biological effect per se [22] and references cited therein), they themselves may contain some active innate or added components that may hinder conclusions on FO direct effects. Furthermore, we avoided the comparison of the effects of two oils (oil of interest and control oil). We applied encapsulated fish oil. In order to avoid degradation of DHA, one capsule containing 1 mL of FO was used for each 3–4 animals (100 µL per animal) and was administered via oral gavage in no longer than 45 s. The remaining FO was discarded and the new capsule was used for next set of animals.

At the end of the treatment, animals were anesthetized (100 mg/kg, Ketamidor, Richter Pharma, Wels, Austria; 16 mg/kg Xylased, Bioveta, a.s.), sacrificed, and perfused with saline solution. One brain hemisphere was taken for fatty acid analysis (*n* = 6 for both control and treated groups). The cortex of the other hemisphere was finely chopped and mixed on ice. Twenty mg of the mixed tissue was taken for protein isolation and another 20 mg for isolation of mRNA (*n* = 4 for Wt and Wt FO; *n* = 6 for 5xFAD and 5xFAD FO). Separate brains were fixed in 4% paraformaldehyde and used for histological staining (*n* = 6).

### 2.3. Lipids Extraction

For the extraction of total lipids, half of the brain was homogenized in 3 mL of chloroform/methanol (1:2 *v*/*v*). After centrifugation, the supernatants were collected and evaporated to dryness. The residues were dissolved in 3 mL of chloroform/methanol (2:1 *v*/*v*) and 3 mL of chloroform/methanol/KCl (4:2:1 *v*/*v*), and centrifuged to eliminate low molecular weight contaminants. After centrifugation, the upper layer was discarded and the lower lipid layers were used for further analysis. During the extraction procedure BHT (2,6-di-tert-butyl-4-methylphenol) was added to the solvents (10 mg/100 mL) to protect lipids from oxidation. 

Phospholipid (PL) fraction was isolated by one-dimensional thin-layer chromatography (TLC) on Silica Gel GF plates (Merck, Darmstadt, Germany) using neutral lipid solvent system of hexane/diethyl ether/acetic acid (87:12:1 *v*/*v*), and methyl esters of PL fatty acids were prepared by transmethylation. 

### 2.4. Phospholipid Analysis

Fatty acids methyl esters were separated by gas-liquid chromatography on a Shimadzu chromatograph GC 2014 (Shimadzu Co., Tokyo, Japan), equipped with a flame ionization detector and GC column Trace TR-Fame (100 m × 0.25 mm ID, film thickness 0.2 μm) (Thermo Fisher Scientific, Waltham, MA, USA). The injection port was set at 220 °C and the flame ionization detector at 260 °C. The oven temperature was programmed at 180 °C for 15 min and then from 180 °C to 240 °C at the heating rate of 1.8 °C/min. The total separation period was 78 min. Individual fatty acid methyl esters were identified by comparing sample peak retention times with PUFA standard mixture (PUFA-2 mix, Supelco, Munich, Germany). Phospholipids FA profiles were expressed as the relative percentage areas of total FA.

### 2.5. Western Blotting

For Western blot analysis, cortical tissue was homogenized in 10 vol (*w*/*v*) of RIPA buffer (50 mM Tris-Cl, pH 7.5, 150 mM NaCl, 1% NP-40, 0.1% SDS, 10 mM EDTA, pH 8.0, 10 mM EGTA, pH 7.2, 0.5% Triton X-100) that contained protease-phosphatase inhibitors (Roche, Basel, Switzerland). Protein concentrations were determined using a Micro BCA Protein Assay Kit (Pierce Biotechnology, Waltham, MA, USA). For extraction of Aβ for western blotting we used RIPA buffer that is stronger than Tris-based buffer and was able to disrupt cellular and vesicular membranes and release their content. Thus, RIPA was able to extract soluble extracellular and cytosolic Aβ forms but also vesicular Aβ species accumulated in the LC-II positive fractions (probably autophagic vesicles), where Aβ monomers are predominantly concentrated [38]. Proteins were loaded in equal amounts of 20 μg per lane, electrophoresed on 12% polyacrylamide gels and transferred onto Immobilon-P membrane (Merck Millipore, Burlington, MA, USA). After blocking (1 h in 5% non-fat dry milk dissolved in Tris-buffered saline/0.1% Tween 20 (TBST)), membranes were incubated overnight at +4 °C, with rat anti-MFSD2a antibody (catalog #10539, Abcam, 1:8000), rabbit polyclonal anti-APP (raised against a 22 amino acid synthetic peptide derived from the carboxy-terminus of β-APP, catalog #51-2700, Zymed) and rabbit monoclonal anti-amyloid beta 42 (raised against a peptide corresponding to amino acids 707-713 of P05067, catalog #700254, Invitrogen, 1:500) in TBST. After washing in TBST, blots were incubated with the horseradish peroxidase (HRP)-conjugated secondary anti-rabbit or anti-mouse antibody (Santa Cruz, Santa Cruz, CA, USA) in TBST for 1 h at room temperature. For loading control, each membrane was re-probed with mouse anti-actin antibody (catalog #A5315, Sigma, St. Louis, MO, USA). The signal was detected using enhanced chemiluminescence (ECL, Amersham GE Healthcare, Little Chalfont, UK) and subsequent exposure on X-ray film. Final analysis was performed using image analysis program ImageQuant 5.0. (GE Healthcare, Little Chalfont, UK).

### 2.6. Real Time Quantitative Polymerase Chain Reaction (RT-PCR)

Total RNA from cortical tissues was isolated using Trizol reagent according to the manufacturer’s instructions (Invitrogen, Carlsbad, CA, USA). Two micrograms of RNA were treated with 2 U of RNase-free DNase I (Thermo Fisher Scientifics, Waltham, MA, USA) and reverse transcribed with a High-Capacity cDNA Archive Kit (Applied Biosystems, Foster City, CA, USA). Polymerase chain reaction (PCR) reactions (15 ng of cDNA, 0.25 μM primers, Power SYBR Green PCR Master Mix from Applied Biosystems) were performed using the ABI 7500 in the default thermal cycling mode. The primer sequences were as follows: mfsd2a, F5′-AGAAGCAGCAACTGTCCATTT-3′, R5′-CTCGGCCCACAAAAAGGATAAT-3′, and b-actin, F5′-TGGACATCCGCAAAGACCTGTAC-3′, R5′-TCAGGAGGAGCAATGATCTTGA-3′. All real time quantitative polymerase chain reaction (RT-PCR) reactions were performed in triplicate, and in at least two independent RT reactions. To confirm the specificity of qPCR reactions, dissociation curves were analyzed at the end of qPCR assay. Relative mRNA levels were calculated using the comparative *Ct* method, and expressed as relative values. 

### 2.7. Plaque Quantification

Following fixation in paraformaldehyde PFA, hemibrains were cryopreserved in graded sucrose solutions (10–30% *w*/*v* sucrose/phosphate buffered saline PBS), frozen in isopentane cooled on dry ice and stored at −80 °C. Three sections (30 μm thick, 120 μm apart) per brain were incubated in 0.01% Thioflavin S (ThioS) solution in 50% ethanol for 8 min at room temperature (RT). The sections were cut between −2 to −2.500 mm from the bregma according to the Allan Mouse Brain Atlas (Allen Institute for Brain Science (2008) available at: http:/alleninstitute.org/) and collected in PBS. Stained sections were washed briefly in 80% and 96% ethanol, rinsed in distilled water and mounted onto glass slides using fluorescent mounting medium (Dako, Santa Clara, CA, USA). Images were captured on an Axio Observer Microscope Z1 using an AxioVision 4.6 software system (Carl Zeiss, Oberkochen, Germany) and digital images were exported to ImageJ (National Institute for Health) for quantitative analysis of each section. To obtain binary image necessary for automatic particle analysis, the maximum entropy thresholding method was chosen. Identified objects after thresholding were individually inspected to confirm the object as a plaque or not in a blinded manner. The average of the individual measurements in the posterior parietal association area (rectangle dimension 1000 × 735 μm) from each mouse was calculated to compare plaque load between the control and FO-supplemented 5xFAD mice.

### 2.8. Statistics

Data were analyzed using Prism program (GraphPad Prism, Software, v.6, La Jolla, CA, USA). The normality of the distributions of values obtained for each group/experimental treatment was determined using the D’Agostino-Pearson normality test. For multiple comparisons two-way analysis of variance (ANOVA) (FO-diet and genotype as factors) with Tukey post hoc test was used when distribution was normal. The Kruskal–Wallis with Dunn’s test was applied when distributions were not Gaussian. The test was considered significant when *p* < 0.05.

## 3. Results

### 3.1. Fish Oil (FO) Differentially Affected n-3 and n-6 Polyunsaturated Fatty Acids (PUFA) Content in the Brains of the Wild-Type (WT) and 5xFAD Mice

**Figure 1:**
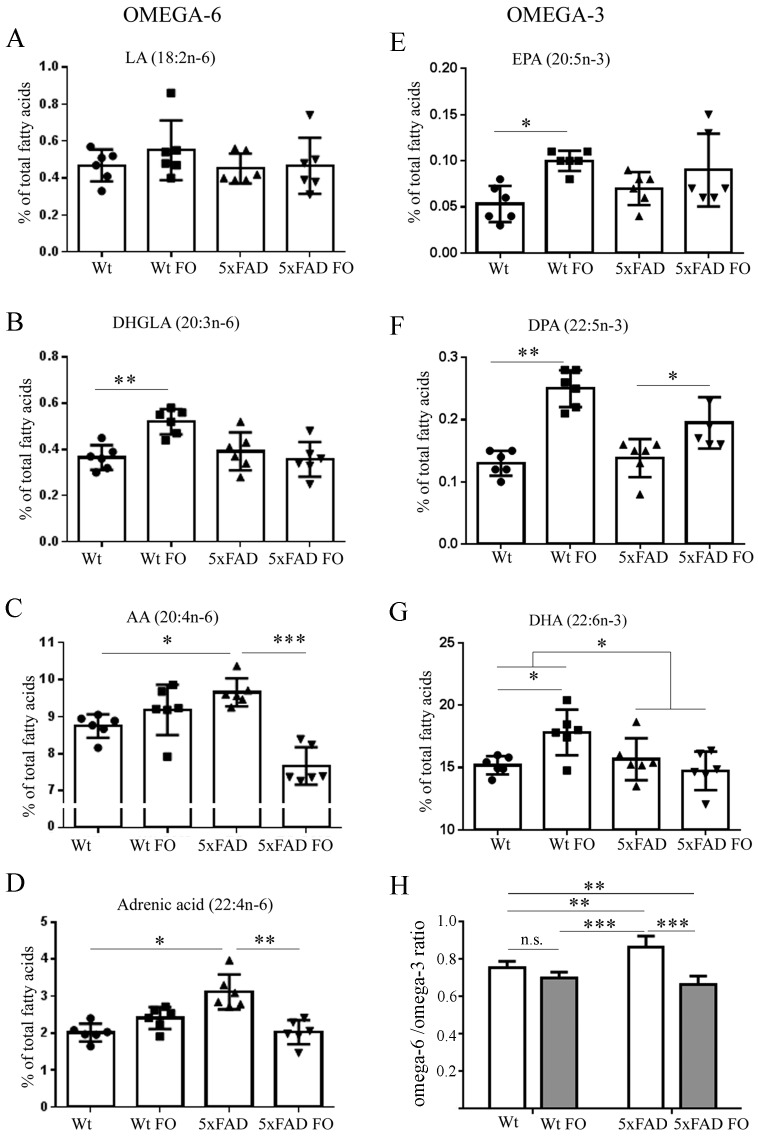
Effects of short-term FO supplementation on *n*-3 and *n*-6 PUFA level in the brain of Wt and 5xFAD mice. (**A**) LA, Linoleic acid (Kruskal-Wallis, *p* = 0.5528); (**B**) DHGLA, dihomo-gamma-linolenic acid (2-w ANOVA, *p*_diet_ = 0.0416, *p*_genotype_ = 0.00222, *p*_interaction_ = 0.0026); (**C**) AA, arachidonic acid (*p*_diet_ = 0.0009_,_ *p*_genotype_ = 0.1496, *p*_interaction_ < 0.0001); (**D**) adrenic acid (Kruskal-Wallis, *p* = 0.0015); (**E**) EPA, eicosapentanoic acid (*p*_diet_ = 0.0032, *p*_genotype_ = 0.7417, *p*_interaction_ = 0.1962); (**F**) DPA, docosapentaenoic acid (*p*_diet_ < 0.0001, *p*_genotype_ = 0.0831, *p*_interaction_ = 0.0224); (**G**) DHA, docosahexaenoic acid (*p*_diet_ = 0.1817, *p*_genotype_ = 0.0483, *p*_interaction_ = 0.0089); (**H**) omega-6/omega-3 ratio (*p*_diet_ < 0.0001, *p*_genotype_ = 0.0569, *p*_interaction_ = 0.0010). Data are shown as a single points per animal (circles, squares or triangles) in each group. Values are presented as mean ± standard deviation (SD). * *p* < 0.05, ** *p* < 0.01, *** *p* < 0.001; n.s., not significant.

As one of the aims of this study was to investigate whether a 3-week-long FO supplementation could change compositions of phospholipids in the brains of Wt and 5xFAD animals, the first step was to determine if there were any differences in the basal level of fatty acids between two genotypes. Lipidomic analysis showed that levels of *n*-3 fatty acids (EPA, docosapentanoic acid DPA and DHA) did not differ between Wt and 5xFAD brains (Figure 1). However, there were significant differences in the levels of *n*-6 fatty acids, as omega-6 arachidonic acid (AA) and adrenic acid levels were higher by 10% (*p* < 0.05) and 55% (*p* < 0.05), respectively, in the brains of 5xFAD compared to Wt brains.

Next, we analyzed the effects of FO dietary supplementation and found significant impact on phospholipids of both groups. The main effect of FO in the Wt group was the elevation of all *n*-3 fatty acids, EPA (2-fold increase, *p* < 0.05), DPA (92%, *p* < 0.01) and DHA (17%, *p* < 0.05), as well as of DHGLA (41%, *p* < 0.01), an *n*-6 fatty acid that has an anti-inflammatory role. In contrast, a dominant effect observed in the 5xFAD group was decrease of the *n*-6 fatty acids, as seen in the reduced concentration of the AA and adrenic acid levels by 21% (*p* < 0.001) and 35% (*p* < 0.01), respectively. Fish oil treatment did not change *n*-3 EPA and DHA levels, but did elevate DPA by 43% (*p* < 0.05).

### 3.2. FO Induced a Similar Pattern of Changes in n-3 and n-6 PUFA Levels in the Livers of Both Genotypes

**Figure 2:**
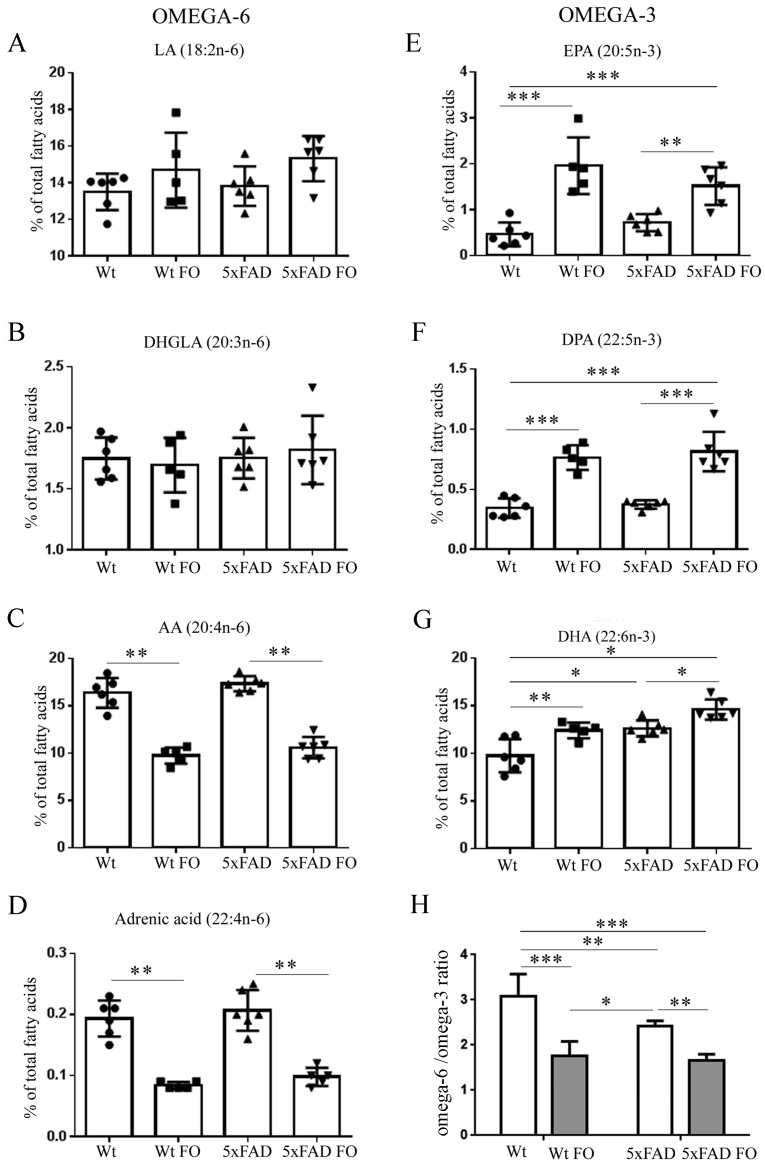
Effects of short-term FO supplementation on *n*-3 and *n*-6 PUFA level in the liver of Wt and 5xFAD mice. (**A**) LA, Linoleic acid (2-w analysis of variance (ANOVA), *p*_diet_ = 0.0219, *p*_genotype_ = 0.3881, *p*_interaction_ = 0.7719), (**B**) DHGLA, dihomo-gamma-linolenic acid (*p*_diet_ = 0.9445, *p*_genotype_ = 0.4869, *p*_interaction_ = 0.509); (**C**) AA, arachidonic acid (Kruskal-Wallis, *p* = 0.0004); (**D**) adrenic acid (Kruskal-Wallis, *p* = 0.0009); (**E**) EPA, eicosapentanoic acid (*p*_diet_ < 0.0001, *p*_genotype_ = 0.5690, *p*_interaction_ = 0.0449); (**F**) DPA, docosapentanoic acid (*p*_diet_ < 0.0001, *p*_genotype_ = 0.3877, *p*_interaction_ = 0.8339); (**G**) DHA, docosahexaenoic acid (*p*_diet_ = 0.0002, *p*_genotype_ < 0.0001, *p*_interaction_ = 0.5067); (**H**) omega-6/omega-3 ratio (*p*_diet_ < 0.0001, *p*_genotype_ = 0.00084, *p*_interaction_ = 0.0410). Data are shown as a single points per animal (circles, squares or triangles) in each group.Values are presented as mean ± SD. * *p* < 0.05, ** *p* < 0.01, *** *p* < 0.001; n.s., not significant.

The next aim was to investigate if 3-week-long FO supplementation alters the composition of phospholipids in the liver tissue (Figure 2). Our analysis revealed that basal levels of individual *n*-3 and *n*-6 fatty acids were not significantly different between Wt and 5xFAD livers. The only exception was DHA, which was higher by 30% (*p* < 0.05) in phospholipids of 5xFAD animals compared to Wts.

Following FO supplementation there were robust increases of EPA (4-fold, *p* < 0.001), DPA (2-fold, *p* < 0.01) and DHA (27%, *p* < 0.05) in the phospholipids of Wt livers. In 5xFAD livers FO also induced the rise of all *n*-3, but the extent of changes in EPA and DHA levels was half of that found in Wts (only double increases of EPA, *p* < 0.01, and 14% of DHA, *p* < 0.01). 

These increases in *n*-3 fatty acid levels were paralleled by a significant, similar extent of reduction, by ~50%, in the levels of *n*-6 AA and adrenic fatty acids in both Wt and 5xFAD genotypes.

### 3.3. Correlation of Individual Omega-6 and Omega-3 Fatty Acids between Liver and Brain

**Figure 3:**
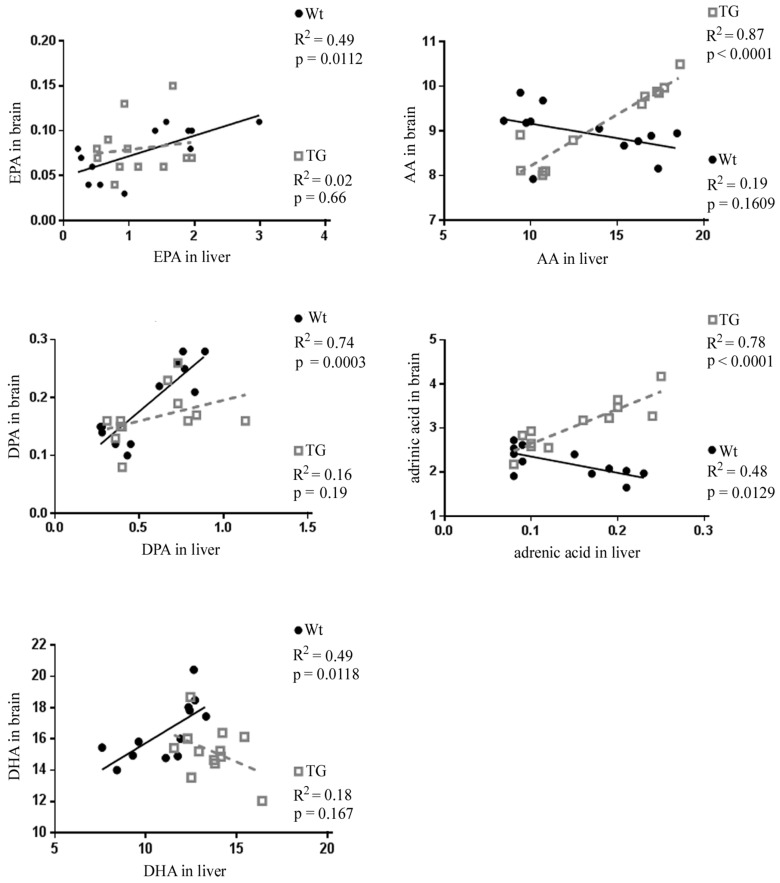
Relationship between the liver and the brain omega-3 and omega-6 fatty acids. Linear regression analysis of EPA, DPA, DHA, AA and adrenic acid levels (percentage of total fatty acids) between the liver and the brain of Wt (black circles) and TG, 5xFAD mice (gray squares). Significant correlation was found for individual *n*-3 fatty acids in Wt animals and for *n*-6 fatty acids in 5xFAD animals.

We then sought to determine if the dietary intake of FO influences an association between liver and brain *n*-3 and *n*-6 fatty acid levels and therefore performed a linear regression analysis (Figure 3). Significant positive correlations were found for EPA (*R*^2^ = 0.49; *p* = 0.0112), DPA (*R*^2^ = 0.74, *p* = 0.0003) and DHA (*R*^2^ = 0.49; *p* = 0.0118) between the liver and brain of Wt animals.

In 5xFAD animals, the increases in EPA, DPA and DHA levels in the liver were not in direct relation with changes observed in the brain (EPA: *R*^2^ = 0.02; *p* = 0.66; DPA: *R*^2^ = 0.16; *p* = 0.19 and DHA: *R*^2^ = 0.18; *p* = 0.167). However, there was a strong and significant correlation between the liver and brain decrease of AA (*R*^2^ = 0.87; *p* = 0 < 0.0001) and adrenic acid (*R*^2^ = 0.78; *p* < 0.0001) in 5xFAD transgenic animals.

Together, these results suggest that livers and brains of both Wt and 5xFAD genotypes were responsive to dietary FO intake. However, a substantial correlation between liver and brain was found only for *n*-3 in Wt animals, and for *n*-6 in 5xFAD.

### 3.4. Effects of FO Supplementation on Total PUFA, Mono-Unsaturated Fatty Acids (MUFA) and Saturated Fatty Acids (SFA) in the Brains and Livers of Wt and 5xFAD Mice

Changes in the quantity of individual fatty acids ultimately have resulted in alterations of total *n*-3 and *n*-6 levels and the *n*-6/*n*-3 ratio in both liver and brain.

In the livers (Table 4), FO supplementation improved *n*-6/*n*-3 ratio in both genotypes as the outcome of increased total *n*-3 (by 43% in Wt, *p* < 0.001, and 23% in 5xFAD, *p* < 0.001) and parallel decrease of total *n*-6 levels (18% in Wt, *p* < 0.05 and 16% in 5xFAD, *p* < 0.05). Total PUFA levels did not change noticeably.

The improvement of *n*-6/*n*-3 ratio was also detected in the brains of both genotypes (Table 5). However, while in Wt animals it was a consequence of significant increase of total *n*-3 (19%, *p* < 0.05), in 5xFAD mice it was a result of a decrease in total *n*-6 level (23%, *p* < 0.001). Accordingly, total PUFA level in the brain after FO supplementation was elevated by 14% (*p* < 0.05) in Wt and reduced by 15% (*p* < 0.05) in 5xFAD animals.

Since analysis of FO composition showed a certain percentage of other fatty acids beside essential PUFAs, especially palmitic, palmitoleic and oleic (Table 3), we also analysed whether FO intake modulates brain and liver tissue content of mono-unsaturated fatty acids (MUFAs) and saturated fatty acids (SFAs). Individual SFA had similar levels in the livers and brains of both Wt and 5xFAD animals. Supplementation with FO, rich in palmitic acid, induced elevation of tissue palmitic acid in both genotypes in the livers by 19% (*p* < 0.05), but total SFA change reached significance only in 5xFAD mice. In the brain, however, palmitic acid level varied differently between genotypes, resulting in the total SFA decrease in Wt (12%, *p* < 0.01) and increase in 5xFAD (9%, *p* < 0.05).

As for MUFA, significant though opposed effects of FO supplementation on individual and total fatty acids were observed mainly in the liver and brain of Wt animals (Table 4 and Table 5).

### 3.5. The Expression of Mfsd2a Transporter Remained Unchanged in the Livers and Brains of Wt and 5xFAD Mice Following FO Supplementation

**Figure 4:**
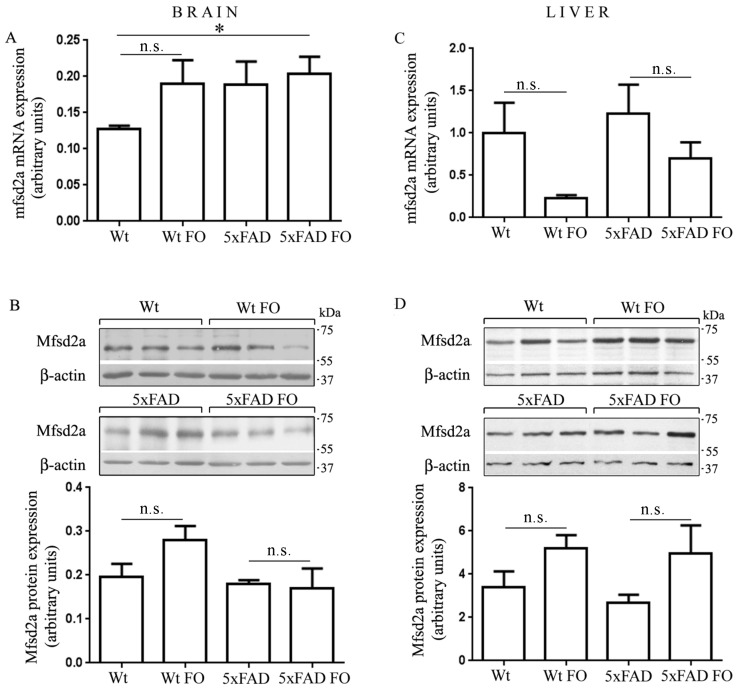
Expression of Mfsd2a in the brain and liver of Wt and 5xFAD mice after fish oil supplementation. The levels of Mfsd2a mRNA and protein were obtained by real-time polymerase chain reaction (RT-PCR) (**A**,**C**) and Western blotting (**B**,**D**), respectively in the brain (left panel) and liver (right panel) of Wt and 5xFAD animals. 03B2-actin served as an internal control of the mfsd2a gene expression and protein load. Data are presented as mean ± SEM (standard error of mean). * *p* < 0.05; n.s., not significant.

Since Mfsd2a was recognized as a specific transporter of *n*-3 DHA in the brain, with lower specificity for palmitic and oleic acid [8], it was of interest to investigate whether FO intake would influence its expression. To evaluate the specificity of Mfsd2a antibody binding, we performed several validation steps, which included induction of a specific band by fasting [28], immunoprecipitation and pre incubation with blocking peptide, all of which confirmed specific immunoreactivity (data not shown). The levels of Mfsd2a mRNA and protein in the brain were not significantly different between Wt and 5xFAD (Figure 4A,B) and no changes were observed following the FO treatment.

In the liver (Figure 4C,D), strong trends of enhanced translation of Mfsd2a mRNA to protein were observed after FO supplementation, but these changes were not significant due to liver individual differences.

### 3.6. FO Supplementation Decreased the Number of Aβ Plaques in the Parietal Cortex of 5xFAD Mice

**Figure 5:**
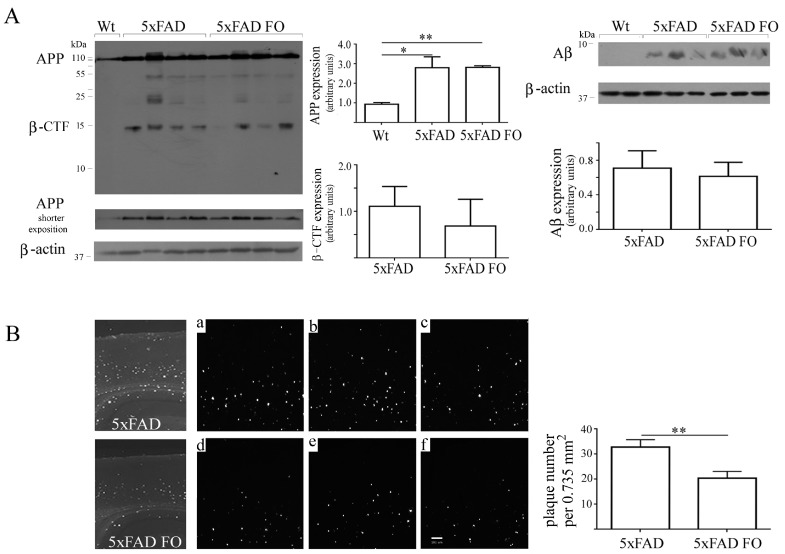
Effects of the fish oil consumption on amyloid precursor protein APP, β-CTF and Aβ expression levels and plaques burden in the cortex of 5xFAD transgenic mice. (**A**) Representative Western blots and quantitative analysis of the full length APP and b-CTF fragment (Zymed, clone 695 that reacts with both human and mouse APP) and its proteolytic product Aβ (Invitrogen, clone H31L2). Quantification of APP band density was performed on less exposed X-ray films. (**B**) Representative low-magnification images of Thioflavin S stained plaques in the brain parietal cortex. Insets show plaque deposits in three different control (**a**–**c**) and FO supplemented (**d**–**f**) animals. Data are presented as mean ± SEM. * *p* < 0.05, ** *p* < 0.01. Magnification, ×5.

As APP processing and accumulation of Aβ forms into plaques is one of the most recognizable hallmarks of AD pathology, we next examined whether three weeks long FO supplementation changed amyloid burden in the cortex of 5xFAD mice (Figure 5). Western blot analysis of APP protein showed that the 5xFAD mouse model expressed stable and significantly higher level of APP full length protein compared to the Wt, and its expression is not altered after FO supplementation (Figure 5A). We also analyzed the expression levels of Aβ using western blotting, and the signal intensity that we obtained was similar to the results previously published in different transgenic models [39,40]. Our data also showed that the FO treatment did not influence amyloid levels considering that the expression of β-CTF and Aβ forms generated by APP proteolytic processing was unchanged. However, analysis of tissue sections stained with Thioflavin S (Figure 5B) demonstrated that FO reduced the number of dense-core plaques by 34% (*p* < 0.01) in the parietal cortex, the brain region that is one of the most vulnerable in AD. 

## 4. Discussion

In this study we have used the 5xFAD transgenic AD model to examine if fish oil treatment, for as short a duration as 3 weeks, may affect fatty acid composition in the brain phospholipids and mitigate the disease in the early stages when extensive plaque deposition was not present yet [37]. Beneficial effects of omega-3 PUFA and its major component DHA have been already demonstrated in several animal models of AD, but the majority of studies (reviewed in [13]) have used considerably longer omega-3 supplementation, lasting from one to more than 18 months. In contrast to some previous studies, we compared the effects of dietary omega-3 PUFA supplementation to the effects of a standard rodent diet that is nutritionally sufficient in lipids, which abolishes the question of whether group differences are a consequence of increased omega-3 levels in the experimental group or low brain omega-3 levels in the control group. Herein, we clearly demonstrated that over the short term, a 3-week-long period of FO supplementation was able to change the phospholipids profile in the brains and livers of both wild-type and 5xFAD transgenic AD mice. While livers of both genotypes responded to FO with the increase of omega-3 and the reciprocal decrease of omega-6 PUFAs, changes in the brains were more complex. We revealed that FO-dependent modulation of the lipid composition may have beneficial effects not necessarily because of the elevation of omega-3 fatty acid levels, but rather due to the reduction of omega-6 fatty acids. 

The distinct pattern of FO-induced changes in FA composition in both Wt and 5xFAD animals is reflective of genotype-associated differences in FA content. Namely, our data revealed significantly higher level of omega-6 arachidonic (AA) and adrenic fatty acids in the brains of 5xFAD mice compared to wild-type littermates, as was found, similarly, in tg2576 AD model [17], Physiologically, AA plays important roles in synaptic signalling, long-term potentiation, learning and memory [41] but its excessive levels could have detrimental consequences through several mechanisms. Firstly, AA can be metabolized to eicosanoids (prostaglandins, leukotriens, and tromboxans) all of which are potent mediators of inflammation, oxidative stress and cerebrovascular dysfunction [41,42]. Secondly, excessive neuronal excitation induced by AA can cause excitotoxicity, and consequently synaptic deficits, network dysfunction, and behavioural deficits [41]. Furthermore, unbound AA released by phospholipase A_2_ from membrane phosphoglicerides can be elongated in a single step to adrenic acid, the third most abundant PUFA in the brain. The specific function of adrenic acid is not yet clear, but it could have some biological activities similar to AA as the abnormalities in AA and adrenic fatty acid levels are implicated in the pathogenesis of Alzheimer’s disease [2]. Thus, the increased level of arachidonic ARA and adrenic acid reported in our study can be critical for neuroinflammation and synaptic dysfunction, AD-associated phenomena prominently present in 4-month-old 5xFAD animals.

By contrast with Wt mice and some AD models [13,21,32], where FO induced a robust augmentation of main omega-3 fatty acids, the major effect of FO supplementation in 5xFAD brains was the reduction of omega-6 AA and adrenic acids. This finding is of great importance as previous data showed the correlation between brain levels of *n*-6 fatty acids, Aβ_1–40_ levels and cognitive performance in transgenic AD mice [43]. The correlation between AA intake and production of its respective derived pro-inflammatory eicosanoids [1], further points to AA and probably adrenic acid as interesting and manageable targets in nutrition-based preventive strategies against AD.

Along with the reduction in omega-6 FA, in 5xFAD brains we also found the FO-induced rise of DPA, one of the omega-3 fatty acids that is an intermediary in the EPA–DHA pathway. The increase of DPA was also significant in the brains of Wt animals. Since DPA was mainly observed as a potential reservoir for EPA and DHA [44], only a few studies have investigated DPA’s independent effects so far, establishing its role as a potent inhibitor of AA metabolism [32,44]. This is substantial as the proinflammatory eicosanoids derived from AA are biologically active in very small quantities [2] and any reduction in their presence may be beneficial for AD pathogenesis. Accordingly, we could presume that FO-induced elevation of DPA in 5xFAD brains is the most favorable outcome at a given circumstance due to its potent AA diminishing effects. 

In our study, surprisingly, the 3-week-long FO treatment induced only a modest increase in DHA levels in the livers of both genotypes and in the brain of Wts, while in the brain of 5xFAD mice the DHA level was unaltered. Dietary conditions significantly influence the content of long-chain PUFAs–especially DHA in the CNS [24]. Unlike our study, numerous studies on other animal models found elevation of DHA after dietary or oral supplementation with DHA in the cortical or hippocampal tissues, especially of females [3,5,6,8,12,15]. One of the reasons for this discrepancy could be slower accumulations of DHA and EPA in the 5xFAD brain. Previously, it was shown that the rate and length of time needed to complete DHA recovery after chronic depletion were specific for tissues, taking 2 weeks in the liver, while cerebral DHA levels do not return to normal until 8 weeks later [45]. It is possible that uptake and delivery of the nervous system are not capable of rapid DHA accumulation in the brain due to transport-related process at the blood–brain barrier that are rate-limiting [45]. This would be in line with unaltered Mfsd2a protein expression found in 5xFAD. Very heterogenic mRNA expression not in accordance with protein level [46] and inconsistent induction of other fatty acid transporters (like FABP5) by FO in transgenic models [21] are already shown, possibly indicating complex mechanisms involved in transporters’ regulation and likely numerous posttranslational modifications. Although we can’t rule out the role of transporters, since livers of 5xFAD did elevate DHA levels making it available for the brain.

The additional explanation for unaltered DHA and EPA levels after dietary supplementation in 5xFAD may be their catabolism to more potent anti-inflammatory and neuroprotective metabolites after entering the brain. The synthesis of resolvins and protectins from DHA and EPA as precursors is increased within 3–4 weeks in blood or peripheral tissue of both human and rodents taking an omega-3 enriched diet, as well as in in vitro studies [47,48,49]. However, this is questionable as the mediators are generated at very small concentrations and the half-life of their original precursors, EPA and DHA, in the brain is much longer [1]. We could also speculate that the disparity of our results regarding DHA elevation may reside in the use of fish oil that normally contains higher amounts of EPA than DHA as a source of omega-3, since the most of published data were obtained following DHA application (reviewed in [50]). Both fatty acids may have distinct functions in the brain and an optimal outcome may be met by FO of different EPA–DHA ratios [24,44]. Notably, FO contains other fatty acids, apart from omega-3 and -6, along with other components (iodine, furan fatty acid, as well as anti-oxidant vitamin E) that can have certain biological effects per se [21,22,23,43]. Although the recent study by Dong et al. (2018) [21] confirmed positive effect of FO administration on DHA level in APP/PS1 transgenic model, results of our study are to a large extent supported by data of Arendash et al. (2007) [43], which have shown no effect of long-term FO feeding on cortical omega-3 fatty acids and soluble/insoluble Aβ within the hippocampus, but suggested benefits of FO, at least in low-risk individuals, through corrections of omega-6 level. Our data strongly suggest that even short-term FO treatment can have positive effects in genetically modified animals that are mediated via the reduction of AA and adrenic omega-6 fatty acids and elevation of DPA, not DHA.

One of the hypotheses underlying the present study was that the short-term FO supplementation in 5xFAD mice would alter AD brain pathological markers, i.e., Aβ aggregations into the amyloid plaques. Indeed, FO supplementation induced a significant reduction in the number of Thioflavin S positive dense-core plaques in the parietal association cortex, the part of the parietal lobe whose role in the development of AD has gained significant attention [4,51]. However, the unaltered level of the full length APP protein and its cleaved Aβ product, along with an unchanged level of DHA incorporation into phospholipids, indicated that potential structural changes in neuronal membranes influencing Aβ generation, may not be the main mechanism of FO beneficial effects in our study. These results are in line with some other studies showing positive effects of omega-3 in transgenic mouse that were not necessarily accompanied by the reduction of brain Aβ [3,5,43,52]. One of the plausible explanations could be that the beneficial effects of DHA in AD can be increased by other specific nutrients found in FO or added to FO-enriched chow as antioxidant vitamin E, which has recently been shown to promote the effects of FO [21]. Other mechanism, suggested by Teng et al., (2015) where DHA effects are not directly attributable to the inhibition of Aβ production, pointed to downstream process of oligomerization through the increase of DHA-mediated less toxic fibrilar vs. more toxic prefibrilar Aβ species. Similar DHA-associated modulation of Aβ aggregation could not be excluded as a potential cause of reduced plaque numbers in our study since short-term FO may likely affect plaque seeding and formation of new plaques than induce the reduction of already existing dense core plaque deposits. Additionally, there were indications that Aβ aggregation into plaques could be hindered by omega-3 immuno-modulatory action. Namely, the 5xFAD model is characterized by a rapid and massive accumulation of Aβ in the brain that induces a robust inflammatory response [37] and sustained microglia activation becomes the predominant feature in 5xFAD model from 4 to 9 months of age [53]. We believe that FO-mediated improvement of the omega-6/omega-3 ratio, induced specifically by a decrease of pro-inflammatory AA and adrenic fatty acids, might stimulate microglia to convert to activation state associated with defence and anti-inflammatory mechanisms [54] in this early phase of AD pathology. This is corroborated with the latest results from our laboratory (data submitted for publication) demonstrating that following a 3-week-long FO supplementation the increased microglia accumulation around plaques acted as a barrier preventing soluble Aβ to bind to dense, compact plaques. Notably, the importance of the microglia barrier is the most prominent at the initial stage of fibrillar amyloid deposition and becomes less effective as plaques grow in size [55]. When a certain critical concentration of Aβ oligomeric form is achieved, plaques form quickly and grow to a mature, stable size within days and that short period may be the time-frame for glial action [56]. As 5xFAD mice before the age of 4 months are in the mild/moderate phase of AD pathology, during the early stage of Aβ accumulation and with no intensive inflammation yet, the 3-week-long FO supplementation could contribute to microglia-mediated activation of protective mechanisms and, thus, attenuate the birth of new plaques and/or modulate the degree of plaque compaction.

There are few limitations of this study. It is possible that longer treatment would be useful in deciphering if 5xFAD mice need more time than Wt littermates to accumulate DHA and EPA and whether it would change Mfsd2a transporter expression. Also, we cannot exclude the possibility that the changes in subcellular localization (lipid rafts) or the distribution of specific phospholipid classes in different brain regions [30] might give valuable information, especially regarding DHA. The studies on the effect of DHA differ in many factors, including animal species and genotype, age and extent of AD pathology, dose, duration of supplementation, route of administration and prior deprivation of omega-3 in the diet, all of which could affect DHA levels and make comparisons between them difficult. These questions are subjects for the future studies. However, this work has revealed several very important facts regarding the potential of specific nutritional factors, even if applied short term, to alleviate the pathogenesis of AD.

## 5. Conclusions

Taken together, our results demonstrated that an early stage of AD pathology in 5xFAD mice model is characterized with elevated levels of omega-6 PUFA, specifically AA and adrenic fatty acid, whose abundance can be efficiently decreased by FO supplementation. Controlling the presence and the ratio of omega-3 and omega-6 PUFAs in the body, especially in the liver and brain, is crucial for optimal brain functioning [2] and can be preventative and therapeutic for a wide range of human diseases including neurodegenerative disorders. Supplementation that usually lasts for months in animal models would require changes in the lifestyle which may be considered as an obstacle in omega-3 wider, preventive usage in the human population. However, we documented that nutritional environmental factors like omega-3 supplementation, even over a duration as short as 1–3 weeks, particularly if applied at the early stage of pathology, could have health-promoting effects despite existing unfavorable genetic factors. 

## Figures and Tables

**Table 1:** Pelletized commercial diet content.

Content	Percentage
Protein	17.2%
Carbohydrate	60.9%
Fat	3.7%
PUFA/SFA	1.3
*n*-3/*n*-6 PUFA	0.05
fiber	5.6%
ash	7.6%
adequate amount of vitamins and minerals	

SFA, saturated fatty acids; PUFA, polyunsaturated fatty acids; *n*-3, omega-3; *n*-6, omega-6.

**Table 2:** Body weights of 3-month-old wild-type (Wt) and 5xFAD mice before (0 week) and during 3-week-long fish oil (FO) supplementation.

Animals	0 Week	1 Week	2 Week	3 Week
Wt	20.22 ± 0.529	20.53 ± 0.629	20.88 ± 0.419	20.80 ± 0.394
Wt FO	20.98 ± 0.627	21.62 ± 0.517	21.40 ± 0.494	21.32 ± 0.346
5xFAD	21.56 ± 0.871	21.96 ± 1.352	21.85 ± 1.155	22.46 ± 1.256
5xFAD FO	21.09 ± 0.681	21.07 ± 0.802	21.03 ± 0.664	21.26 ± 0.969

**Table 3:** Fatty acid composition of fish oil (% *w*/*w* of total fatty acids).

FA Saturation	Number of Carbons	Common Name	Percentage
SFA	16:0*n*	palmitic	22.90
	18:0*n*	stearic	2.23
MUFA	16:1*n-*7	palmitoleic	11.90
	18:1*n-*9	oleic	10.25
	18:1*n-*7	vaccenic	4.54
*n-*6	18:2*n-*6	linoleic	1.67
	20:3*n-*6	dihomo-gama-linolenic	0.29
	20:4*n-*6	arachidonic	1.62
	22:4*n-*6	adrenic	1.78
*n-*3	20:5*n-*3	EPA	25.51
	22:5*n-*3	DPA	1.82
	22:6*n-*3	DHA	15.49

SFA, saturated fatty acids; MUFA, mono-unsaturated fatty acids; *n*-6, omega-6 polyunsaturated fatty acids (PUFA); *n*-3, omega-3 polyunsaturated fatty acids; EPA, eicosapentaenoic acid; DPA, docosapentaenoic acid; DHA, docosahexaenoic acid.

**Table 4:** SFA, MUFA and PUFA in the livers of WT and 5xFAD control and FO supplemented mice.

FA (%)	Wt	Wt FO	5xFAD	5xFAD FO
Palmitic acid (16:0)	22.33 ± 1.48	26.56 ± 1.47 ^a2^	20.92 ± 0.89	24.87 ± 2.10 ^a,^^b1^
Stearic acid (18:0)	22.67 ± 2.61	21.57 ± 1.57	21.57 ± 1.43	21.00 ± 1.59
SFA	45.01 ± 3.09	48.13 ± 1.73	42.48 ± 1.74	45.87 ± 2.63 ^b^
Palmitoleic acid (16:1*n*-7)	1.04 ± 0.30	0.83 ± 0.08	0.74 ± 0.16	0.66 ± 0.08 ^a^
Oleic acid (18:1*n*-9)	8.40 ± 0.48	7.47 ± 0.59 ^a^	7.33 ± 0.46 ^a1^	6.56 ± 0.72 ^a2^
Vaccenic acid (18:1*n*-7)	3.15 ± 0.54	2.21 ± 0.19 ^a1^	2.58 ± 0.51	2.01 ± 0.43 ^a1^
MUFA	12.59 ± 1.12	10.52 ± 0.67 ^a1^	10.66 ±1.07 ^a^	9.23 ± 1.13 ^a2^
*n*-6	31.84 ± 1.81	26.21 ± 2.79 ^a1^	33.13 ± 1.41	27.95 ± 1.12 ^a2,b2^
*n*-3	10.56 ± 1.89	15.14 ± 1.35 ^a2^	13.73 ± 0.73 ^a1^	16.95 ± 1.48 ^a2,b2^
PUFA	42.40 ± 3.23	41.35 ± 2.05	46.86 ± 2.00 ^a^	44.90 ± 1.99

SFA, saturated fatty acids; MUFA, mono-unsaturated fatty acids; *n*-6, omega-6 polyunsaturated fatty acids (PUFA); *n*-3, omega-3 polyunsaturated fatty acids. Values are presented as mean ± SD. Significantly different from Wt group: ^a^ *p* < 0.05, ^a1^ *p* < 0.01, ^a2^ *p* < 0.001. Significantly different from 5xFAD group: ^b^ *p* < 0.05, ^b1^ *p* < 0.01, ^b2^ *p* < 0.001.

**Table 5:** SFA, MUFA and PUFA in the brains of Wt and 5xFAD control and FO supplemented mice.

FA (%)	Wt	Wt FO	5xFAD	5xFAD FO
Palmitic acid (16:0)	28.49 ± 1.46	25.83 ± 2.57 ^a^	27.00 ± 1.85	29.11 ± 3.17
Stearic acid (18:0)	25.92 ± 3.0	22.94 ± 0.99	24.29 ± 2.20	26.89 ± 2.53
SFA	54.41 ± 22.69	48.77 ± 4.36 ^a1^	51.29 ± 1.64 ^a^	56.01 ± 4.36 ^b^
Palmitoleic acid (16:1*n*-7)	0.46 ± 0.15	0.49 ± 0.13	0.37 ± 0.09	0.47 ± 0.10
Oleic acid (18:1*n*-9)	14.32 ± 1.52	15.02 ± 0.75	14.64 ± 1.67	14.05 ± 2.38
Vaccenic acid (18:1*n*-7)	3.85 ± 0.26	4.71 ± 0.54 ^a^	4.19 ± 0.77	3.93 ± 0.61
MUFA	18.62 ± 1.87	20.40 ± 0.31 ^a^	19.21 ± 2.33	18.45 ± 2.86
*n*-6	11.60 ± 0.49	12.66 ± 0.99 ^a^	13.62 ± 0.85 ^a2^	10.51 ± 0.66 ^a1,b2^
*n*-3	15.26 ± 0.71	18.17 ± 1.84 ^a1^	15.88 ± 1.72	15.03 ± 1.56
PUFA	26.97 ± 1.06	30.83 ± 2.75 ^a1^	29.50 ± 2.46 ^a^	25.54 ± 2.16 ^b^

SFA, saturated fatty acids; MUFA, mono-unsaturated fatty acids; *n*-6, omega-6 polyunsaturated fatty acids (PUFA); *n*-3, omega-3 polyunsaturated fatty acids. Values are presented as mean ± SD. Significantly different from Wt group: ^a^ *p* < 0.05, ^a1^ *p* < 0.01, ^a2^ *p* < 0.001. Significantly different from 5xFAD group: ^b^ *p* < 0.05, ^b2^ *p* < 0.001.
